# Women and the Marriage Laws

**Published:** 1892-08

**Authors:** 


### WOMEN AND THE MARRIAGE LAWS.

Perhaps the most startling thing which occurred at the meeting of the Women's Liberal Federation in England, was the acceptance of a motion in favor of Dr. Hunter's divorce bill, now before Parliament, which not only allows to the woman relief from the marriage tie for the same cause as is held sufficient in the case of a man, but so extends the law that four years' desertion becomes a reason for the dissolution of a marriage. It was always supposed that women were the upholders of the old ecclesiastical idea of wedlock. It is a sign of the times, and points to an approaching complete revolution in the English marriage laws, and that at the instance of the women themselves.

